# Spinal accessory nerve transfer for shoulder abduction has no benefit over supraclavicular exploration and nerve grafting in brachial plexus birth injury: a systematic review

**DOI:** 10.3389/fped.2024.1426105

**Published:** 2024-12-19

**Authors:** Dhruv Mendiratta, Rohan Singh, George Abdelmalek, Krittika Pant, Alice Chu, Aleksandra McGrath

**Affiliations:** ^1^ Department of Orthopaedic Surgery, Rutgers New Jersey Medical School, Newark, NJ, United States; ^2^ Department of Clinical Sciences, Umeå University, Umeå, Sweden; ^3^ Department of Surgical and Perioperative Sciences, Umeå University, Umeå, Sweden

**Keywords:** brachial plexus birth injury, peripheral nerve, surgery, outcome, nerve graft, nerve transfer

## Abstract

**Introduction:**

Brachial plexus birth injury (BPBI) has an incidence of 0.9 per 1,000 live births in the population. Techniques for repair classically include supraclavicular exploration and nerve grafting (SENG) and more recently nerve transfer, namely of the spinal accessory nerve (SAN) to the suprascapular nerve (SSN) to improve functional outcomes such as glenohumeral abduction and external rotation. This systematic review was conducted to evaluate whether spinal accessory nerve transfer produced significantly better outcomes for shoulder abduction in BPBI.

**Methods:**

A search was conducted using Preferred Reporting Items for Systematic Reviews and Meta-Analysis Individual Patient Data guidelines. Standardized comparisons were made using the Mallet Score for shoulder abduction.

**Results:**

10 full-text articles with itemized patient outcome measures were selected. 110 patients were identified with 51 patients in the SENG group and 59 patients in the SAN transfer group. The mean shoulder abduction Mallet score in the SENG group was 3.50 ± 0.84, while the mean Mallet score in the SAN transfer group was 3.58 ± 0.77, which displayed no significant differences (*p* = 0.9012). There was no significant relationship between the age at time of surgery and post-operative Mallet scores for shoulder abduction after SENG (*p* = 0.3720).

**Discussion:**

Our systematic review found that there was no difference observed in post-operative outcomes of shoulder abduction when comparing SAN transfer and nerve grafting. Continued support for nerve grafting lies in the argument that it incorporates the patient's native neuroanatomy and allows for sensory reinnervation.

## 1 Introduction

Brachial plexus birth injury (BPBI) has an incidence of 0.9 per 1,000 live births in the United States population (1), but a large proportion of these injuries do not require treatment. For the approximately 30% of patients who do not achieve spontaneous recovery, treatment options include the use of secondary surgeries or microsurgery. Secondary procedures encompass muscle transfers and osteotomies among other orthopedic techniques. Microsurgical repair predominantly involves nerve grafting, however the advent of nerve transfers provided a novel alternative surgical approach. Nerve transfers rely on reanimating the injured nerves with a healthy donor nerve, rather than exploring the injured roots of the brachial plexus and repair with a nerve graft (2). Nerve transfers were originally performed in adult patients with traumatic brachial plexus injuries, before gaining popularity for treatment of BPBI. Nerve transfers have been reported as increasingly popular procedures in complicated BPBI, namely late presentations, incomplete recoveries, or failure of primary reconstruction (3).

According to Narakas’ classification, there are four distinct presentations of brachial plexus birth injury, namely upper-Erb's (C5-C6), extended Erb's (C5-C7), total palsy without Horner syndrome (C5-T1), and total palsy with Horner Syndrome (C5-T1) (4). In upper-Erb's brachial plexus injury, there is a loss of or decreased shoulder abduction, flexion, external rotation, and elbow flexion. It has been reported that deficits in shoulder function would likely remain when following a conservative treatment approach, even with spontaneous recovery of elbow flexion (5). The two main targets for reconstruction of shoulder abduction are the suprascapular nerve for initiation of abduction by the supraspinatus muscle, and the axillary nerve, which supplies the deltoid muscle. The suprascapular nerve is estimated to be involved in 98% of BPBI patients (6). Thus, treatment approaches include supraclavicular exploration and nerve grafting (SENG) and spinal accessory nerve (SAN) nerve transfer to the suprascapular nerve (SSN). While distal nerve transfers have been increasingly utilized, supraclavicular exploration and grafting remains a viable option in surgical management of brachial plexus injury, based on both clinical experience and experimental work (7–10). SENGs potential benefit for abduction over SAN is targeting not only supraspinatus but also deltoid. Double nerve transfers (radial to axillary nerve and SAN), which address both important shoulder abductors have been pioneered for BPBI patients, however data on their outcomes remains limited, compared to established SAN (11, 12).

There have been some studies comparing types of microsurgical repair in outcomes for BPBI. Tse et al. found no statistically significant difference in shoulder external rotation when comparing nerve grafting and SAN transfer. However, this study ultimately concluded that the type of surgery should be based on the individual lesion, and that future comparisons would benefit from randomization of treatment groups (13). In contrast (14), found cervical root grafting to result in worse outcomes for shoulder function and to be associated with a two-fold higher frequency of secondary shoulder surgery when compared to nerve transfer (14). Another variable factor in the treatment of BPBI is the age at primary surgery. There are findings that support both early surgery (within 6 months of life) (15) and studies showing that somewhat delayed surgery does not necessarily result in worse outcomes (16).

There is a paucity of studies that have compiled existing data to determine if differences exist among the various modalities of microsurgical repair. The goal of this systematic review was to evaluate whether spinal accessory nerve transfer produced significantly better outcomes for shoulder abduction in BPBI compared to supraclavicular exploration and nerve grafting (SENG) and whether age was a significant factor using the currently available literature.

## 2 Materials and methods

### 2.1 Literature search

Preferred Reporting Items for Systematic Reviews and Meta-Analysis Individual Patient Data (PRISMA-IPD) guidelines were employed for this study (17). A systematic search of the literature was conducted using Pubmed, Cochrane, Web of Science, and the Cumulative Index to Nursing and Allied Health Literature (CINAHL) databases. Specific search terms including “brachial plexus”, “injury”, “palsy”, “nerve plexus”, “upper plexus”, “pediatric” and “surgery” were used. The complete collection of Boolean searches is provided in the Appendix. From the initial set of articles, duplicates were removed, followed by an abstract and full text screening. In these screenings to build the preliminary database, English text studies on brachial plexus surgery in pediatric patients were identified.

### 2.2 Inclusion and exclusion criteria

The exclusion criteria for these studies were as follows: (i) studies that were not full text; (ii) studies classified as commentaries, review papers or editorials; (iii) studies that were non-human or had less than 3 participants; (iv) studies which had full texts that were inaccessible through institutional or open access forums. For completion, the references of all selected articles were cross-checked. If these articles were not previously included and fulfilled the inclusion criteria, they were included in the preliminary database. The review of this initial database was conducted by a group of authors under the supervision of the senior authors. Every subsequent stage of the process was conducted by the study authors, with any disputes always resolved by the senior authors.

From this preliminary database, articles were then screened for relevance to this study's specific objective. Studies with non-pediatric cases, traumatic injury or with secondary surgeries were excluded. Only studies which investigated patients who received spinal accessory nerve transfers or treatment with supraclavicular exploration and nerve grafting were included. Studies with participants who received these nerve transfers were then divided into groups based upon the type of outcome measures used to evaluate shoulder abduction. These included Mallet scores, active range of motion (ROM) in degrees, angular degree of true glenohumeral external rotation, modified Gilbert and other customized classification schemes. To standardize comparisons for this systematic review, the scope was further narrowed to studies with only patients who received Mallet scores (18) used to evaluate shoulder abduction or scoring systems (Gilbert and Active ROM) that could be converted to Mallet scores. Then, studies that did not perform a primary SAN transfer were excluded. A similar systemic search and narrow was used to filter literature selected for a subset of data for patients undergoing supraclavicular nerve exploration and grafting. Thus, the overall inclusion criteria were accessible, full-text articles on primary non-traumatic pediatric brachial plexus injuries treated with primary SAN transfer or SENG and provided shoulder abduction evaluation through Mallet scores or Gilbert scores and active range of motion.

### 2.3 Data extraction

Data extracted from articles in the preliminary database included number of patients, patient characteristics, follow-up, outcomes following surgery and information about any secondary procedures. This preliminary database was used to determine which studies were relevant to the systematic review, and more specific data was collected. This included the number of patients, the specific details of the SAN transfer, the outcome measure used for shoulder abduction, whether the data was individual or grouped and the numerical result. For the articles used in the present analysis, the primary outcome of interest was Mallet score to evaluate shoulder abduction postoperatively. The patient age at the time of the procedure was extracted for each individual patient. Data extraction was conducted by the authors, as mentioned above, and any disagreements about the relevant data was resolved through prompt discussion with the principal investigators. Authors of articles that did not include comprehensive individual data for patient ages and Mallet scores were contacted for additional information. If these authors did not respond or could not provide additional data, these studies were excluded from the present analysis.

### 2.4 Score conversion

Studies that included Active range of motion (ROM) or Gilbert shoulder abduction scores were converted to Mallet scores. Active ROM scores were converted to Mallet scores based on the guidelines published by Mallet (19). Gilbert scores were converted to Mallet scores using the following categories: Angles of shoulder abduction less than 30 degrees were Mallet grade 2, angles between 30 and 90 degrees were Mallet grade 3, and angles greater than 90 degrees were Mallet grade 4 (18, 19).

### 2.5 Statistical analysis

We used regression analysis to model (i) the relationship between age at the time of surgery and Mallet scores for shoulder abduction received either after SAN or SENG, and (ii) the treatment effect of SAN or SENG on the Mallet scores, controlling for the patients’ age, and the model is as follows:

(i) Mallet score = Intercept + Age * β
(ii) Mallet score = Intercept + Age * β1 + treatment (SAN or SENG) * β2 + Age * treatment * β

Since the estimated regression parameters (β) have large variances among the different papers, we adopted a new statistical method called “iFusion learning” to combine results from all the literature in the preliminary database (20). The main concept in using this type of statistical analysis is to give weight to each study according to their variance. In doing so, studies whose estimated parameters that have smaller standard errors will have a larger effective impact in the overall analysis. For the second model, we did not consider variation between studies and assumed the treatment effect of SAN or SENG will be the same across all studies because of the small sample size.

The Wilcoxon rank test is a nonparametric statistical test, which we used to compare whether there was a significant difference in resulting Mallet scores between the two treatment groups, SAN transfer and SENG. The null hypothesis is that the mean of the two samples are the same. The Wilcoxon rank test was appropriate for our dataset of discrete data points because it does not rely on the assumption that the dataset is normal.

## 3 Results

**Figure 1:**
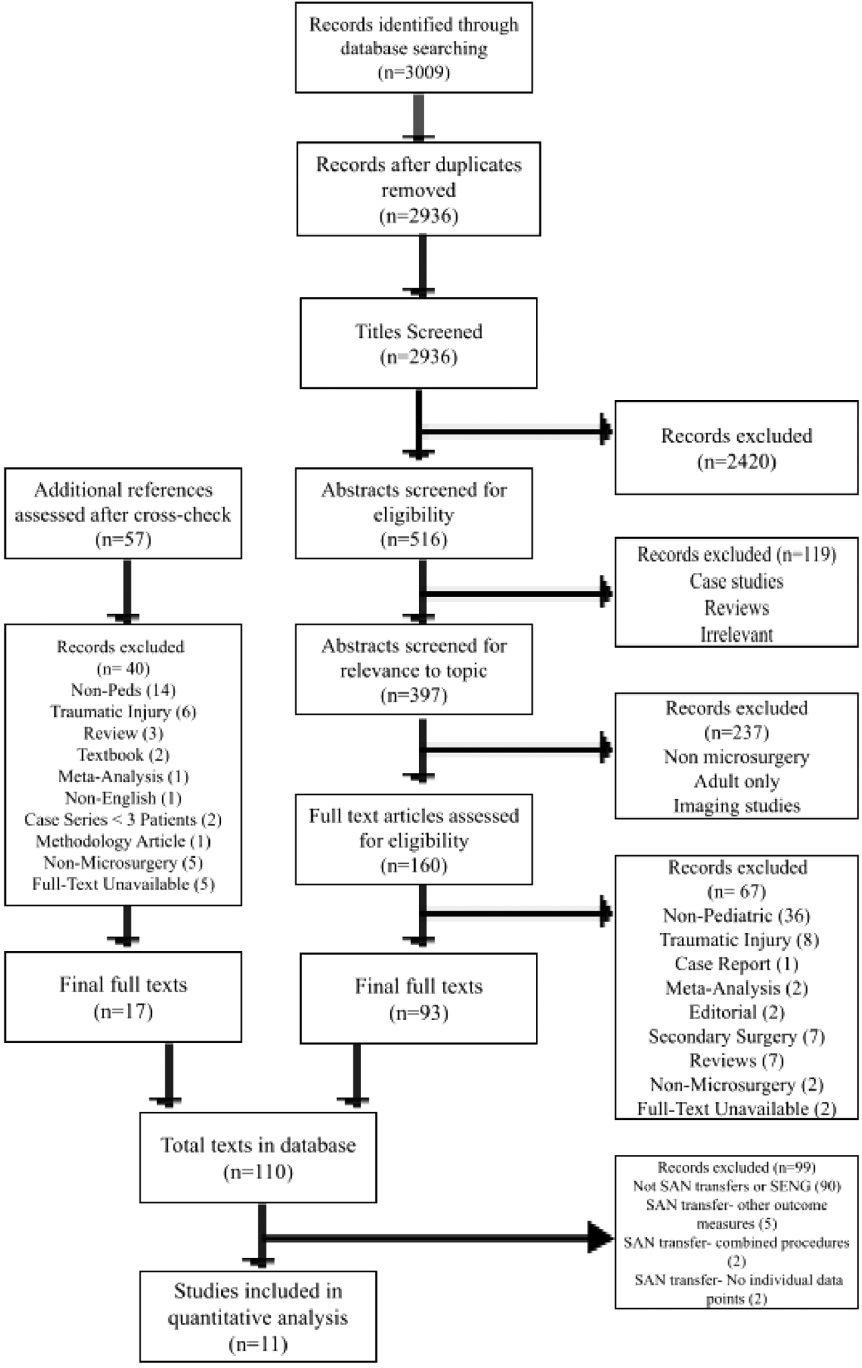
PRISMA study selection.

After removing duplicates, 2,936 studies were identified through PubMed, Cochrane, Web of Science, CINAHL databases. 160 full-text articles were advanced to full-text review. After eligibility and relevance screening, 93 full-text articles and 17 full texts from a snowball search of reference lists were then used to build a database of 110 papers that were studies of primary nerve procedures for BPBI. Of these, 10 had mention of SAN transfer or SENG with outcome measures. The PRISMA outline is detailed in Figure 1.

**Figure 2:**
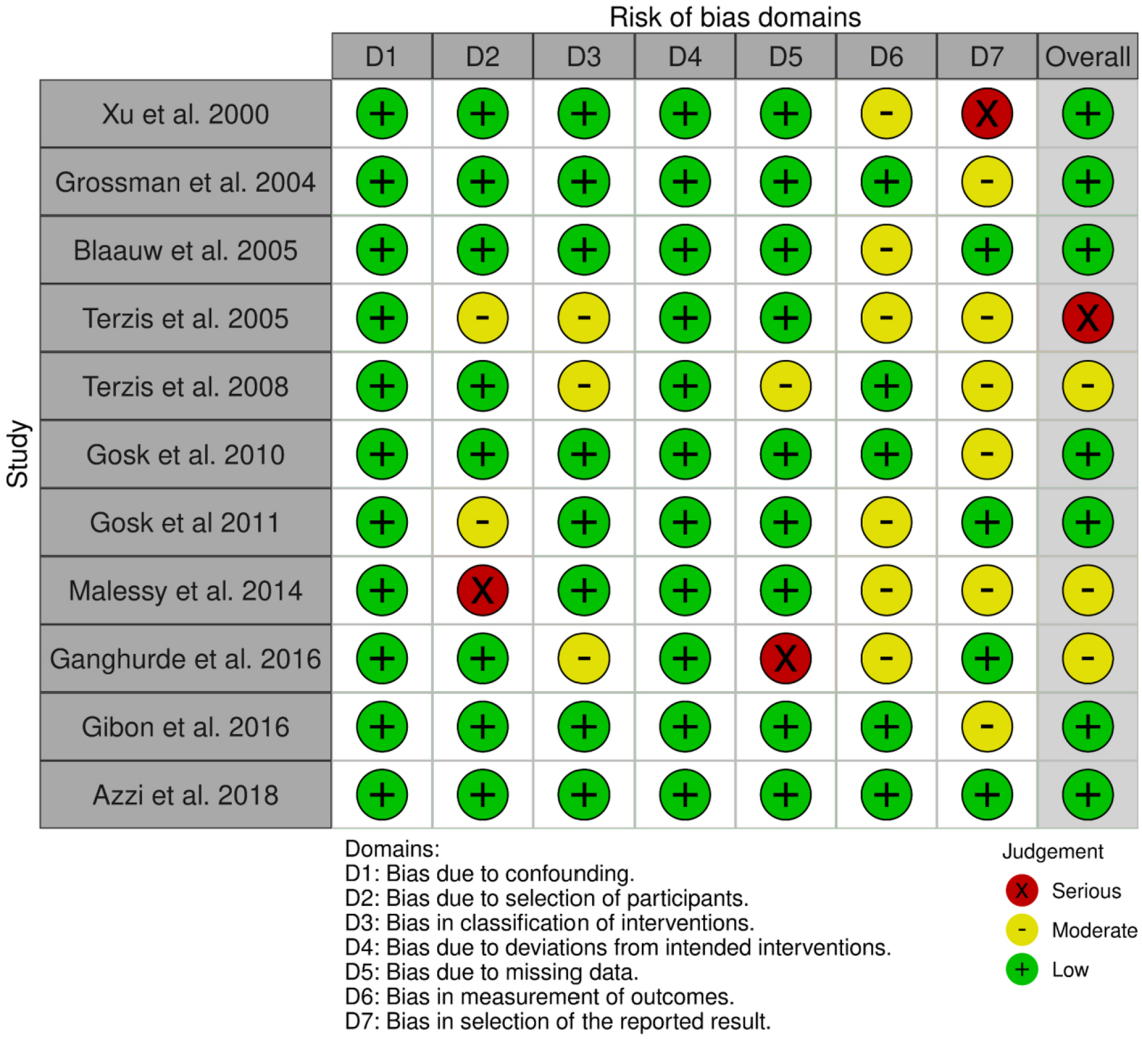
ROBINS-I risk of bias assessment.

The Risk of Bias in Non-Randomized Studies- of Interventions (ROBINS-I) tool was used to assess the risk of bias in the selected studies. The studies primarily had low to moderate risk of bias, with one paper having serious overall bias (Figure 2). The domains of serious bias across these studies included selection of participants, missing data, and in selection of the reported results.

There were 110 patients included in the subsequent analyses (51 patients in the SENG group, 59 patients in the SAN transfer group). The total mean age of all patients was 10.20 ± 10.13 months. The total mean Mallet score for shoulder abduction was 3.54 ± 0.80. The specific characteristics from each of the 11 individual included studies are included in Table 1.

**Table 1:** Characteristics of included studies.

Study	Outcome used	Groups compared	General findings
32-Xu et al. (21). Different methods and results in the treatment of obstetrical brachial plexus palsy. *Journal of reconstructive microsurgery*, 16(6), 417–422. doi:http://dx.doi.org/10.1055/s-2006-947147	Mallet Score- shoulder abduction and external rotation, elbow flexion	Conservative treatment, neurolysis, nerve transfer and grafting SAN to SSN transfer in 1 patient. - 10 total patients	Results from the nerve transfer and grafting group were found to be superior to neurolysis and conservative treatment. 70% of patients in this group achieved “excellent” or “good” results (Mallet scores of 5 or 4 respectively). There were no significant differences found between scores in the conservative treatment and neurolysis group.
112-Malessy and Pondaag (5). Neonatal brachial plexus palsy with neurotmesis of C5 and avulsion of C6: supraclavicular reconstruction strategies and outcome. The Journal of bone and joint surgery. American volume, 96(20), e174. doi:http://dx.doi.org/10.2106/JBJS.M.00547	MRC Grade Active and Passive ROM Mallet scores- shoulder function	Transfer of C6 anterior root filaments or the entire C6 nerve to C5, Grafting from C5 to the anterior division of the superior trunk SAN to SSN transfer in 29 patients. - 34 total patients	- Shoulder abduction: Mallet scores of 4 in 14/17 patients who received intraplexal transfer of C6 to C5, and in 8/17 patients who recieved grafting from C5 to the anterior division of the superior trunk. However, no statistically significant difference was found.
181-Grossman et al. (22). Shoulder function following late neurolysis and bypass grafting for upper brachial plexus birth injuries. Journal of hand surgery (Edinburgh, Scotland), 29(4), 356–358. doi:http://dx.doi.org/10.1016/j.jhsb.2004.03.008	Modified Gilbert System	N/A- all patients underwent a microsurgical neurolysis using intraoperative neurophysiologic monitoring SAN to SSN transfer in 1 patient. - 11 total patients	- Median increase of 3 grades in the shoulder function score. - 6/11 cases had an improvement of 3 or more grades.
75-Terzis and Kostas (23). Outcomes with suprascapular nerve reconstruction in obstetrical brachial plexus patients. Plastic and reconstructive surgery, 121(4), 1267–1278. doi:http://dx.doi.org/10.1097/01.prs.00,00,305537.74910.bf	MRC scale Mallet scores	N/A-Case reports: Case 1 (SAN-SSN transfer with interposition nerve grafts), Case 2 (SAN- SSN transfer), Case 3 (SSN nerve repaired from the C5 proximal stump using nerve grafts) SAN to SSN transfer in 1 patient. - 3 total case reports	Case 1: “excellent” shoulder abduction and external rotation (follow up of 2 years). Case 2: 90 degrees of shoulder abduction, 90 degrees of external rotation (follow up of 9 years). Case 3: 90 degrees of shoulder abduction, “excellent” shoulder external rotation (follow up of 4 years).
147-Azzi et al. (11). Restoration of shoulder motion using single- versus dual-nerve repair in obstetrical brachial plexus injury. Journal of neurosurgery. Pediatrics, 21(5), 511–515. doi:http://dx.doi.org/10.3171/2017.11.PEDS17493	ROM (degrees) against gravity	Single nerve repair (interpositional nerve grafting of the upper trunk), Dual nerve repair (additional neurotization of the SAN to SSN) SAN to SSN transfer in 10 patients. - 18 total patients	- No statistical differences between postoperative shoulder abduction and external rotation ROMs or ROMs gained between the two groups. - Mean postoperative abduction ROM gained was 83.2 degrees after single nerve repair and 89.0 degrees after dual nerve repair.
130- Ghanghurde et al. (24). Distal transfers as a primary treatment in obstetric brachial plexus palsy: a series of 20 cases. The Journal of hand surgery, European volume, 41(8), 875–881. doi:http://dx.doi.org/10.1177/1753193416663887	Modified MRC Mallet score	N/A- SAN to SSN transfer for shoulder function restoration, Oberlin transfer for elbow function restoration SAN to SSN transfer in 17 patients. - 20 total patients	- Three patients lost to follow up among remaining patients: - Mean shoulder abduction Mallet score of 3.2. - Mean shoulder external rotation Mallet score of 3.1.
209-Terzis and Kostas-Agnantis (25). Reconstruction of Shoulder Abduction and External Rotation in Obstetrical Brachial Plexus Palsy Patients. Semin Plast Surg. 19. 10.1055/s-2005-867110.	MRC with intermediate grades Mallet scores	N/A- Case presentations: Case 1 (supraclavicular plexus was explored- SSN was reconstructed from the C5 proximal stump), Case 2 (SSN to SAN, posterior cord was reconstructed from the proximal stump of the C6 root, C6 root was used for lateral cord reconstruction), Case 3 (SSN was directly neurotized by SAN, proximal stump of the C5 root was used to reconstruct the distal C8 and TI roots, additional nerve grafts from C5 roots) SENG in 1 patient - 3 total cases	- Case 1: Shoulder abduction and external rotation Mallet scores of 4 (postoperative). - Case 2: 90 degrees shoulder abduction, 30 degrees external rotation (2 years of follow up). - Case 3: 80 degrees shoulder abduction, 15 degrees external rotation (3 years of follow up).
91- Gosk et al. (26). Comparison of the results of surgical treatment after direct neurorrhaphy and reconstruction with sural nerve grafts in perinatal brachial plexus lesions. Folia neuropathologica, 48(4), 270–275.	Gilbert scale	Direct neurorrhaphy, Reconstruction with sural nerve grafts SENG in 5 patients - 14 total patients	- Shoulder abduction Mallet scores of 0, 4, 4, 4, 4. - Shoulder abduction Mallet scores of 5, 4, 4, 3, 4, 4, 4, 3. - Superior results were shown with direct neurorrhaphy as compared to reconstruction with sural grafts.
125- Gibon et al. (27). Isolated C5-C6 avulsion in obstetric brachial plexus palsy treated by ipsilateral C7 neurotization to the upper trunk: outcomes at a mean follow-up of 9 years. The Journal of hand surgery, European volume, 41(2), 185–190. doi:http://dx.doi.org/10.1177/1753193415593493	Modified MRC Mallet scores	N/A- All patients were treated with a total ipsilateral C7 neurotization to the upper trunk SENG in 7 patients - 10 total patients	- Three children lost to follow up. - Active shoulder abduction ranged from 35 degrees to 120 degrees. - Muscle strength for shoulder abduction ranged from 3 to 4 with an average Mallet score of 3.1. - Results ultimately showed good restoration of shoulder abduction strength, but relatively poor restoration of active shoulder external rotation.
58-Blaauw et al. (28). Hypoglossal nerve transfer in obstetric brachial plexus palsy. Journal of plastic, reconstructive & aesthetic surgery: JPRAS, 59(5), 474–478. doi:http://dx.doi.org/10.1016/j.bjps.2005.07.013	***Results section shows “all six children received this procedure…as a result of the transfer of the accessory nerve to the suprascapular nerve”	SENG in 2 patients	
96-Gosk et al. (29). Neurolysis of the conducting neuroma-in-continuity in perinatal brachial plexus palsy—evaluation of the results of surgical treatment. Folia neuropathologica, 49(3), 197–203.	Gilbert scale Gilbert and Raimondi's scale Al-Qattan's scale Modified MRC (Omer and Dellon)	N/A- External neurolysis, external and internal neurolysis, external neurolysis of upper and lower trunk + extra anatomical direct reconstruction of spinal nerve C7 with cervical plexus, external neurolysis of middle and lower trunk + reconstruction of upper trunk with grafts. SENG in 8 patients.	- Upper injury: 1 child had good shoulder and elbow function. - Upper-middle injuries with neuroma-in-continuity in upper trunk: good function achieved in 83.3% of patients. - Total injuries with neuroma-in-continuity localized in the upper trunk: good shoulder function in 50% of those examined. - Best results with neurolysis are when the developed neuroma-in-continuity is localized in the upper trunk and a normal motor response is obtained via electrical stimulation.

### 3.1 Effect of patient age on shoulder abduction outcomes

The mean age of patients in the SENG group was 10.89 ± 11.33 months. There was no significant relationship between the age at time of surgery and post-operative Mallet scores for shoulder abduction after SENG. This is both before (age estimate = −0.01089; *p* = 0.3720) and after (age estimate = −0.04838; *p* = 0.2405) the iFusion method was used. The mean age of patients in the SAN transfer group was 9.69 ± 9.22 months. Similarly, there was no relationship between the age at the time of surgery and post-operative Mallet scores for shoulder abduction after SAN transfer, both before (age estimate = −0.02854; *p* = 0.17) and after (age estimate = −0.1004; *p* = 0.3983) the iFusion method was used.

### 3.2 Comparing outcomes after SENG and SAN transfer

The mean shoulder abduction Mallet score in the SENG group was 3.50 ± 0.84, while the mean Mallet score in the SAN transfer group was 3.58 ± 0.77. There was no treatment effect of SAN or SENG on the Mallet scores, when controlling for patients’ ages at the time of surgery (treatment estimate = −0.03428; *p* = 0.8930). There is no difference between Mallet scores for shoulder abduction after SAN transfer or SENG treatment (W = 1,258; *p* = 0.9012).

## 4 Discussion

Our systematic review found that there was no difference observed in post-operative outcomes of shoulder abduction when comparing SAN transfer and nerve grafting. The traditional treatment for brachial plexus birth injury was nerve grafting. A shift toward nerve transfer in pediatric patients occurred after increased use of nerve transfer in adults with brachial plexus palsy (30, 31). The advantages of nerve transfer as compared to nerve grafting are a quicker delivery of regenerating nerve fibers to the target end organ resulting in earlier reinnervation, direct motor-to-motor nerve coaptation, and less extensive surgical dissection (30, 32). Previous studies have found success with SAN to SSN transfers in BPBI patients, with one study citing recovery of active external rotation in 71.5% of their patients (33, 34). Showed 94.4% “good” outcomes for active shoulder movements in their BPBI patients after SAN transfer, which was found to be better as compared to only 66.7% “good” outcomes with C5 root neurotization (34). However, it is not clearly established that nerve transfer produces superior outcomes compared to nerve grafting. Continued support for nerve grafting lies in the argument that it incorporates the patient's native neuroanatomy and allows for sensory reinnervation (30).

Although there is limited literature on brachial plexus birth injury, prior studies have explored SAN transfer to the suprascapular nerve in older subjects with brachial plexus palsy. For instance (35), presented a study with older patients treated with SAN transfer as part of a dual nerve transfer for traumatic brachial plexus palsy achieving functional recovery in 77.2% of participants with average shoulder abduction of 55 degrees (35). Similarly, in a study by (36), excellent or good functional outcomes for shoulder abduction were achieved in almost 60% of adult patients (36). The efficacy of SAN transfer in older patients provided an opportunity to explore the technique in brachial plexus birth injury. Most recently (30), conducted a direct comparison of nerve grafting and SAN transfer for patients with BPBI and proposed that better outcomes with nerve transfers in the BPBI patients compared with traumatic BPI may be due to a smaller distance of regeneration. However, Smith et al. additionally noted that longer periods of follow-up following surgery may be needed to establish that SAN transfer outcomes remain superior to nerve grafting over time (30). Considering these findings, our systematic review investigated the potential differences in functional outcomes between SAN transfers and nerve grafting. Although SAN transfers have been documented to be advantageous to nerve grafting, our review elucidates no differences observed in shoulder abduction between the two treatments.

It has been established that nerve reconstruction will result in improved use of the arm, as compared to no intervention in BPBI, if there is no recovery of biceps function by 4 months of age (37). Furthermore, increasing delay in recovery of biceps function indicates worse outcomes in global shoulder functioning (38). Wilson et al. developed a decision algorithm based on maternal and neonatal factors to aid clinicians in determining whether surgical intervention is warranted in cases of BPBI. With a high positive predictive value of 94% (39), this algorithm is crucial in progressing toward a universal evidence-based treatment paradigm which will provide the best outcomes after surgery. However, there is still debate regarding individual patient characteristics, such as age at surgery, and their association with post-operative outcomes. Our study showed no significant relationship between age at the time of surgery and post-operative outcomes for shoulder abduction after SAN transfer. In our study, a possible reason for why age is not associated with postoperative outcomes, regardless of the surgical technique used, may be due to the smaller limb length of children. Nerve grafting is proximal while nerve transfer is a distal technique. This is thought to contribute to better outcomes in nerve transfer due to a shorter distance of reinnervation, as stated above. However, this may not translate to significant discrepancies in post-operative outcomes in children due to a smaller difference in anatomic location between the proximal and distal sites, especially for shoulder reconstruction.

### 4.1 Limitations

In gathering data from numerous studies across a large time period, our biggest limitation was creating a final database of studies with standardized outcomes. In evaluating BPBI, there is variation across the outcome measures that are reported, follow-up periods after surgery and ages at the time of assessment. As per (40), the results of the iPluto world-wide consensus survey supported the use of active ROM and Mallet scores (40), which was the basis for using Mallet scores in our study. In this study, we did not look at shoulder external rotation as an outcome measure (4). Noted that 20% of children have a discrepancy between Mallet scores for shoulder abduction and external rotation (18). It would be fruitful to devise future studies where both shoulder external rotation and abduction are examined to investigate whether this discrepancy exists and what it may indicate for global shoulder function in each patient. Similarly, once more evidence becomes available, future studies ought to explore efficiency of double transfers (SAN and radial to axillary nerve) vs. SENG. Although we were able to convert outcomes measured through Gilbert scoring and ROM, there were many studies that did not report individual outcomes. Efforts were made to contact authors for a more comprehensive dataset, but this was unsuccessful. This, in conjunction with studies that were excluded due to heterogeneity in outcome measures, decreased the statistical power of our review.

The nature of the retrospective analysis renders it susceptible to bias, which we sought to evaluate using the ROBINS-I assessment. The included studies were non-randomized, had patients who were operated on by surgeons with varying skill levels, and had different follow-up periods. Missing data was present in some studies and there may have been inaccuracies in recorded data.

It is difficult to make conclusions about age or the difference between outcomes without the gold standard of randomized, double-blind controlled trials. The decision to use a nerve transfer vs. nerve grafting is often made during surgical exploration with a treatment strategy uniquely designed for each individual patient. However, there are several barriers to conducting such a trial due to ethical considerations, difficulty in obtaining parental consent, issues with recruitment and the current variation in management (41). With the acknowledged need to better standardize treatment strategies for BPBI, there should be a focus on reporting data in a manner conducive to inclusion in future systematic reviews.

## 5 Conclusion

In patients with BPBI, there is no difference in postoperative shoulder abduction outcomes when comparing SAN transfer and SENG. There was also no association between age at the time of surgery and postoperative shoulder abduction outcomes after either procedure.

## Data Availability

The raw data supporting the conclusions of this article will be made available by the authors, without undue reservation.

## Appendix

**Search Terms:**

PubMed search (January 2020):

((brachial plexus pals*[MeSH] OR brachial plexus pals*[tiab] OR brachial plexus injury[MeSH] OR brachial plexus injury[tiab] OR upper plexus[tiab] OR nerve plexus injury[tiab] OR brachial nerve injury[tiab] OR brachial plexus surgery[tiab] OR brachial nerves injury[tiab] OR tbpi[tiab] or traumatic bpi[tiab]) AND (infant[MeSH] OR child[MeSH] OR adolescent[MeSH] OR children[MeSH] OR child[tiab] OR child*[tiab] OR adolescent[MeSH] OR adolescent[tiab] OR infant[tiab] OR baby[tiab] OR young[tiab] OR youth[tiab] OR kid[tiab] OR kids[tiab] OR pediatric[MeSH] OR pediatric[tiab] OR pediatrics[MeSH] OR pediatrics[tiab]) AND (surg*[MeSH] OR surg*[tiab]))

Cochrane, Web of Science, and CINAHL search (January 2020):

((brachial plexus pals* OR brachial plexus pals* OR brachial plexus injury OR brachial plexus injury OR upper plexus OR nerve plexus injury OR brachial nerve injury OR brachial plexus surgery OR brachial nerves injury OR tbpi or traumatic bpi) AND (infant OR child OR adolescent OR children OR child OR child* OR adolescent OR adolescent OR infant OR baby OR young OR youth OR kid OR kids OR pediatric OR pediatrics) AND (surg*))
